# Morphometric Analysis of Nonsclerosed Glomeruli Size and Connective Tissue Content during the Aging Process

**DOI:** 10.1100/2012/845046

**Published:** 2012-05-02

**Authors:** Vesna R. Stojanović, Ivan D. Jovanović, Sladjana Z. Ugrenović, Ljiljana P. Vasović, Vladimir S. Živković, Miodrag V. Jocić, Braca K. Kundalić, Miljana N. Pavlović

**Affiliations:** ^1^Department of Anatomy, University of Niš, Faculty of Medicine, Dr Zoran Djindjic Boulevard 81, 18000 Niš, Serbia; ^2^Institute of Transfusiology, Military Medical Academy, Crnotravska 17, 11000 Belgrade, Serbia

## Abstract

Number of sclerotic glomeruli increases during the aging process. Consequently, majority of remained nonsclerosed glomeruli become hypertrophic and some of them sclerotic, too. The aim of this study was to quantify the size and connective tissue content of nonsclerosed glomeruli and to evaluate the percentage of hypertrophic ones in examined human cases during the aging. Material was right kidney's tissue of 30 cadavers obtained during routine autopsies. Cadavers were without previously diagnosed kidney disease, diabetes, hypertension, or any other systemic disease. Tissue specimens were routinely prepared for histological and morphometric analysis. Images of the histological slices were analyzed and captured under 400x magnification with digital camera. Further they were morphometrically and statistically analyzed with ImageJ and NCSS-PASS software. Multiple and linear regression of obtained morphometric parameters showed significant increase of glomerular connective tissue area and percentage. Cluster analysis showed the presence of two types of glomeruli. Second type was characterized with significantly larger size, connective tissue content, and significantly lower cellularity, in relation to the first type. Such glomeruli might be considered as hypertrophic. First type of glomeruli was predominant in younger cases, while second type of glomeruli was predominant in cases older than 55 years.

## 1. Background

Glomerular aging changes include progressive decline in number of normal, intact glomeruli, increase in number/percentage of globally sclerotic glomeruli especially in outer cortical regions, increase in number of abnormal glomeruli with shunts between afferent and efferent arterioles in juxtamedullary area, focal or diffuse thickening of the glomerular basement membrane, and, increased volume of mesangial matrix [1]. More recent researches showed that decreased number of glomeruli in older individuals is related with significantly lower birth weight, which predisposes their kidneys to the conditions of aging [2].

Glomerulosclerosis represents sign of nephron loss and glomerular equivalent of scarring. Under the age of 40 up to 10% of glomeruli are completely sclerosed [3]. By the eighth decade 10–30% of glomeruli are sclerosed. Outer cortex glomeruli are especially affected. Development of sclerosis is the consequence of mesangial matrix increase, glomerular basement membrane thickening, and free intraglomerular anastomoses formation [4]. This leads to compensatory hypertrophy of remained, especially juxtamedullary glomeruli, caused by glomerular hyperfiltration and increase of intracapillary pressure [3]. The increase in size of such glomeruli is pathophysiologically significant and their hyperperfusion leads them into further sclerosis, first segmental and then global [5, 6].

These aging changes cause glomerular filtration rate decrease of 8–10 mL/min per decade, and reduction in cortical renal mass in elderly [1]. Decline in renal function during the aging is not clinically significant until acute or chronic illness further impairs renal reserve. However, this decline in renal function has very important implications for renal transplantation. Transplants from older donors now account for 14% of all first cadaver transplants. Impaired functional reserve in the renal allografts from the older donors can suggest that they might be inadequate for maintaining function after transplantation. Additionally, uninephrectomy in older donor may hasten aging process in the remaining kidney due to consequent hyperfiltration. This can have deleterious effect on kidney function with increase of intraglomerular pressure, flow, and increased filtration rate per one nephron [7]. Although globally sclerotic glomeruli can disappear during the life, then their percentage cannot be used as reliable parameter of nephron loss during the aging process [8]. The aim of our study is to investigate the size and connective tissue content of patent, nonsclerosed glomeruli during the aging process. In such way we would indirectly estimate the presence of hypertrophic glomeruli in cases of different age, independently of the globally sclerotic glomeruli percentage. This might be helpful in the further decisions about older donors' kidney usage in the transplantation purposes.

## 2. Material and Methods

Material was right kidney's tissue of 30 cadavers, obtained during routine autopsies at the Department of forensic medicine at Medical Faculty in Niš. Their age ranged from 25 to 85 years. Cadavers were without previously diagnosed kidney disease, diabetes, hypertension, or any other systemic disease. During autopsy kidney damage was not observed, too. Tissue specimens were fixed in 10% buffered formalin for 24 hours and then embedded in paraffin. Tissue was then cut into 5 *μ*m thick sections and routinely stained with Mallory trichrome stain. Histological slices were analyzed under 400x magnification. Images of histological slices were captured with digital camera (5 megapixels resolution). Glomerular tufts were analyzed with ImageJ software (http://rsbweb.nih.gov/ij/) which was spatially calibrated with object micrometer (1 : 100). Glomerular tuft area (*A*_*G*_), perimeter (*B*_*G*_), Feret's diameter (*D*_*F*_), total number of cells per glomerular area unit (*N*_*n*_), and glomerular connective tissue area (*A*_CT_)) were measured. Glomerular images were additionally processed for connective tissue area measurement. Glomerular tuft image was first manually selected by polygonal selection tool and extracted from the other parts of histological slice image. Selection of its connective tissue, which was green stained on Mallory trichrome stained sections, was performed by “color-based tresholding” option. Its application was based on green colored sample of glomerular tuft image. Afterwards, only green stained parts of glomerular tuft remained on image, which was further converted into binary image. Binary image was used for connective tissue area measurement. Green colored samples were taken at three different localizations in each glomerular tuft image. Connective tissue area was measured for each sample. Average connective tissue area was then calculated from three obtained values for each glomerular tuft. Glomerular connective tissue percentage was obtained from the ratio between glomerualar connective tissue area and total glomerular area. Seven cortical, seven columnar, and seven juxtamedullary glomeruli, selected by unbiased method, were analyzed per one case. Additionally, no more than seven globally sclerotic glomeruli were also analyzed per one case. So, according to Li et al. [9], the number of evaluated glomeruli per one case ranged from 21 to 28, in dependence of the number of observed sclerotic glomeruli. Sclerotic glomeruli served as positive control during morphometric analysis. Totally, 743 (114 sclerotic and 629 morphologically nonsclerosed) glomeruli were analyzed in all 30 cases. Average values of morphometric parameters were calculated for each of all 30 evaluated cases.

### 2.1. Statistical Analysis

Statistical analysis was performed with NCSS-PASS software (http://www.ncss.com/) and included linear regression, multiple regression, cluster analysis (*k*-means method), and Chi-square test. One-way ANOVA was used for the comparison of more than two groups, followed by Tukey-Kramer post hoc test. In cases where data did not have normal distribution Kruskal-Wallis one-way ANOVA was used for the comparison of more than two groups. Obtained results were additionally analyzed with Dunn's post hoc *z*-test. Student's *t*-test was used for the comparison of two groups parameters and Mann-Whitney *U* test was when data did not have normal distribution. We considered that data did not have normal distribution in cases in which morphometric parameters had skewness higher than +1 or −1. Cluster analysis was performed twice during this study. Firstly it was used for the classification of glomeruli into types according to their morphometric characteristics and secondly for the classification of the evaluated human cases into the groups, according to the percentage of obtained types of glomeruli and their age.

## 3. Results

**Figure 1:**
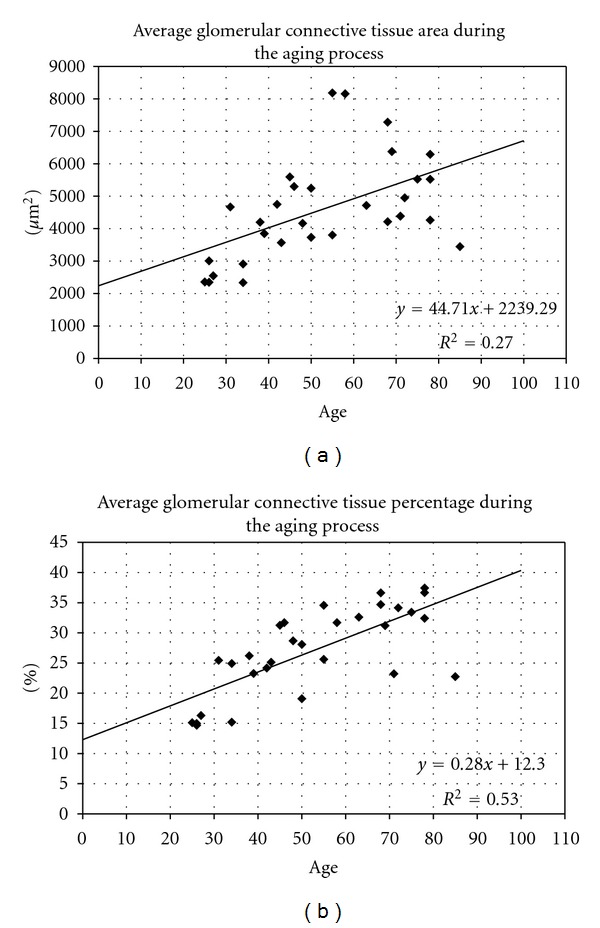
Average connective tissue area of nonsclerosed glomeruli during the aging process (a); average percentage of connective tissue in nonsclerosed glomeruli during the aging process (b).

Results of morphometric analysis of all analyzed human cases are presented in Table 1. Linear regression analysis showed significant positive correlation between the age on one and, glomerular connective tissue area (*F*(1,28) = 10.45, *P* = 0.003, adjusted *R* square 0.246), and percentage of glomerular connective tissue (*F*(1,28) = 31.06, *P* < 0.001, adjusted *R* square 0.509), on the other side (Figure 1). Adjusted *R* square of the connective tissue area was very low because beside the age, glomerular size significantly influenced on average glomerular connective tissue area, too. That is why we additionally performed multiple regression with age and average total glomerular area as independent and average connective tissue area as dependent variable. This analysis, also, showed significant positive correlation between the age and average total glomerular area on one and the average glomerular connective tissue area (*F*(2,27) = 27.39, *P* < 0.001) on the other side. Adjusted *R* square in that case increased up to 0.645, which was significantly higher than the same obtained during linear regression. Average number of cells per glomerular area unit showed decrease during the aging process, but this decrease was not significant. Other morphometric parameters (total glomerular area, perimeter, and Feret's diameter) did not significantly change during the aging process.

Table 2 presents the morphometric parameters of the male and female cases. Average glomerular area (*t* = 2.74, df = 28, *P* = 0.01), average glomerular perimeter (*t* = 3.29, df = 28, *P* = 0.003), and average glomerular Feret's diameter (*t* = 3.07, df = 28, *P* = 0.005) were significantly higher in male than in female cases, which pointed to the conclusion that male glomeruli were significantly larger than the female ones. Average number of cell per glomerular area was opposite to the latter which was cited as being significantly higher in female than in male cases (*t* = 3.04, df = 28, *P* = 0.005). However, in spite of these differences, average glomerular connective tissue area (*t* = 0.73, df = 28, *P* = 0.47) and average glomerular connective tissue percentage (*t* = 1.38, df = 28, *P* = 0.18) were not significantly different between male and female cases, respectively. 

**Figure 2:**
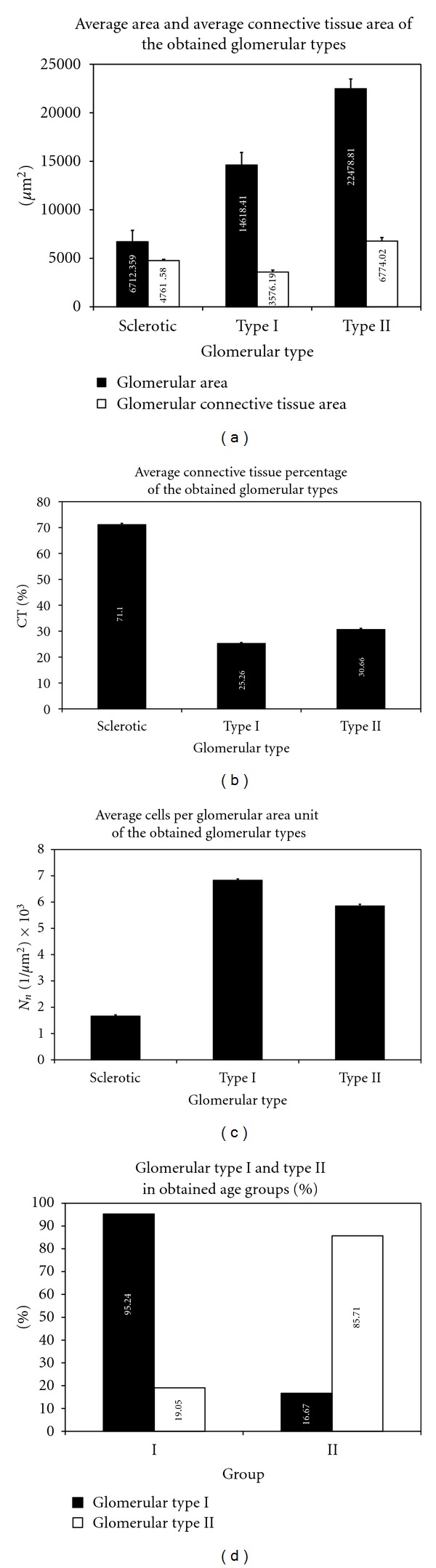
Morphometric characteristics of sclerotic, type I (normal) and type II (hypertrophic) glomeruli in evaluated cases; (a) average glomerular area and average glomerular connective tissue area; (b) average glomerular connective tissue percentage; (c) average number of cells per glomerular area; (d) percentage of type I (normal) and type II (hypertrophic) glomeruli in obtained age groups.

Morphological analysis showed the presence of three different types of glomeruli: normal, hypertrophic, and globally sclerotic. Globally sclerotic glomeruli contained the highest amount of connective tissue and sparse cells inside them. Hypertrophic and normal glomeruli were very similar morphologically. However, hypertrophic ones were larger, with higher amount of connective tissue and with lower number of cells per one glomerulus than normal glomeruli. So, morphologically, only globally sclerotic glomeruli were clearly distinguishable, while differences between normal and hypertrophic glomeruli were discrete. So, in order to establish the percentage of normal and hypertrophic glomeruli in each of 30 evaluated human cases, cluster analysis of 629 morphologically nonsclerosed glomeruli was performed. Their average connective tissue area, average percentage of connective tissue, and average number of cells per glomerular area unit were used as classification parameters. Results showed the presence of two significantly different types of glomeruli. First type included 341 and the second type included 288 glomeruli. Their morphometric characteristics and morphometric characteristics of globally sclerotic glomeruli are presented in Table 3. One-way ANOVA and Tukey-Kramer multiple-comparison test were performed and they showed that first type was characterized with significantly higher average glomerular area (*F*(2,740) = 256.73, *P* < 0.001) (Figure 2(a)), average perimeter (*F*(2,740) = 324.02, *P* < 0.001), and average Feret's diameter (*F*(2,740) = 332.22, *P* < 0.001) than sclerotic, but lower than glomeruli of the second type. Kruskal-Wallis one-way ANOVA and Dunn's test showed that average connective tissue area was in this type of glomeruli significantly lower than in sclerotic and second type glomeruli (*H* = 368.36, df = 2, *P* < 0.001) (Figure 2(a)). Average percentage of glomerular connective tissue (Figure 2(b)) showed the same trend as average connective tissue area (*F*(2,740) = 3690.24, *P* < 0.001). Average number of cells per glomerular area unit was significantly the highest in the first, lower in the second type, and the lowest in the globally sclerotic glomeruli (*F*(2,740) = 1352.47, *P* < 0.001) (Figure 2(c)). Generally, second type of glomeruli had glomerular connective percentage higher than 30% and might be considered as hypertrophic, while the first type glomeruli can be considered as normal. Chi-square test did not show significant (*H* = 1.37, *n* = 629, df = 2, *P* = 0.50) predomination of cortical, columnar, or juxtamedullary glomeruli in obtained glomerular types. Finally, we calculated the percentage of the first and the second type glomeruli in each of all 30 evaluated cases. According to obtained percentages and the age, which were used as classification parameters for cluster analysis, two groups, both with 15 cases, were differentiated. Mann-Whitney *U* test showed that first group cases were significantly younger than the cases of the second group (*z* = −3.65, *P* < 0.001). Their age ranged from 27 to 50 years (Md = 38), while the age of the second group cases ranged from 55 to 75 years (Md = 68). Median percentage of the first type glomeruli was 95.24% (CI. 80.95%–100%) in the first group cases, while it was 19.05% (CI. 9.52%–23.81%) in the second group cases (Figure 2(d)). This difference was significant according to the Mann-Whitney *U* test (*z* = −4.52, *P* < 0.001). Opposite to the latter, second type glomeruli median percentage was significantly higher (*z* = −3.86, *P* < 0.001) in the second group cases (Md = 85.71%; CI. 76.19%–90.48%) than in the first group cases (Md = 16.67%; CI. 4.76%–38.10%) (Figure 2(d)). So, from this, it can be concluded that cases older than 55 years were characterized predominantly with the presence of hypertrophic glomeruli, while normal glomeruli predominate in cases which were younger than 55 years. 

## 4. Discussion

Etiology and pathogenesis of global glomerulosclerosis remain unclear. There are two major possible pathways which might lead to glomerulosclerosis. The first includes immunologic mechanisms with circulating or in situ immune complexes formation [10]. The second includes nonimmunologic mechanisms such as hemodynamic factors. According to Brenner hypothesis, dysregulation of afferent and efferent arteriole leads to increased glomerular plasma flow, which results in increased intracapillary pressure and subsequent glomerular injury with mesangial matrix accumulation. Consequent vascular adaptations help to preserve glomerular filtration rate by producing the state of hyperfiltration and hyperperfusion of the remained nephrons. Finally, this state leads to the glomerular hypertension and hypertrophy with subsequent further mesangial matrix accumulation and eventually focal segmental glomerulosclerosis [1].

Glomerular size varies with different degrees of glomerulosclerosis. In the early phases of sclerosis, glomerular size increases. Afterwards, glomerular tuft shrinks back to normal and then to smaller size. Obsolete glomeruli represent very late stage of glomerulosclerosis. They are nonfunctional and many of them disappear from the kidney. The increase in glomerular size is pathophysiologically significant and represents compensatory response to the loss of functional glomeruli and consequent hyperperfusion of remained patent glomeruli [5]. Glomerular size might be very important in detection of glomerular hypertrophy which represents early phase in the development of glomerulosclerosis. It increases during childhood. However, studies which were focused on adults and elderly gave conflicting results and did not completely define the influence of aging on glomerular size. In study by Nyengaard and Bendtsen [11] the age was inversely correlated with the number of glomeruli (nephrons). Size of the glomeruli was, also, inversely proportional to the age and kidney weight. So, their results confirmed assumption that humans lose glomeruli during the aging process. In their study, Li et al. showed significant increase in glomerular size during the aging. They considered that the reason for that is age-related loss of nephrons number, which causes disorders of glomerular hemodynamics, rise of capillary pressure, and consequent glomerular hypertrophy [9, 12]. Tan et al. in their morphometric study of kidney tissue obtained from older and young donors observed significant increase of globally sclerotic glomeruli percentage in older donor group versus in younger donors group. According to them [13] nonsclerosed glomeruli showed a trend of increased volume in older than in young donors group. This resulted in significant increase of filtration surface area and single nephron ultrafiltration coefficient. This sclerosing glomerulopathy led to consequent glomerulopenia and compensatory hypertrophy with adaptive hyperfiltration of nonsclerosed glomeruli. In our case, linear regression analysis did not show significant change of nonsclerosed glomeruli size during the aging process. However, significant increase of glomerular connective tissue content and the percentage of connective tissue were observed. Classification analysis showed the presence of two types of glomeruli in all evaluated cases. Second type of glomeruli was significantly larger than the first type. These glomeruli had significantly higher connective tissue content and significantly lower number of cells per glomerular area unit. They probably present hypertrophic glomeruli which are in the initial phase of sclerosis. According to the Rodríguez-Puyol [4] the number of cells in hypertrophic glomeruli is increased. In our case, these glomeruli indeed had higher number of cells per glomerulus. But, their area increase was much higher than the increase of number of cells. This resulted in significant decrease of the number of cells per glomerular area unit. Percentage of such hypertrophic glomeruli was significantly higher than the first type of glomeruli in the second group of cases which were older than 55 years. This is in accordance with findings of Tan et al. that allografts from the older deceased donors (age above 55 years) are characterized with marked glomerulopenia, reduction in renal volume, prevalent glomerulosclerosis and interstitial fibrosis and hypertrophy of remaining nonsclerosed glomeruli. They also observed reduction of coefficient of filtration and compensatory single nephron ultrafiltration coefficient increase. Finally, all these changes resulted in reduced glomerular filtration rate of older donors' allograft [13–15].

Therefore, previous researches were mainly focused on the evaluation of glomerular size, the number of the renal corpuscles, and the number of sclerotic glomeruli during the aging process [8, 11]. However, the fact that sclerotic glomeruli may disappear during the aging [8] and that glomerular size and number may vary in relation to the body size makes these parameters unreliable in the evaluation of the functional capacity of the kidney which might be used as allograft during the transplantation. Researches which tried to quantify connective tissue content in nonsclerosed glomeruli during the aging process were very rare. We consider that such quantification might represent more reliable method for the establishment of glomerular hypertrophy among nonsclerosed glomeruli population during the aging. The percentage of such glomeruli with higher connective tissue content, which we established during our research, might be more important for the functional capacity of the potential kidneys allografts, especially the ones which originate from the donors older than 50 years.

## 5. Conclusion

So, results of morphometric analysis pointed to the significant increase of connective tissue content in nonsclerosed glomeruli during the aging process. Cases older than 55 years contain predominantly hypertrophic glomeruli which have significantly higher connective tissue content and lower number of cells per glomerular area unit. Such glomeruli probably are in the initial phase of glomerulosclerosis and their predomination in elderly might be responsible for the impairment of their renal functional reserve, which further can indirectly suggest that allografts from the older donors might be inadequate for maintaining renal function after the transplantation.

## Figures and Tables

**Table 1:** Morphometric parameters average values of nonsclerosed glomeruli in all 30 evaluated cases.

Case	Gender	Age	*A_G_* (**μ**m^2^)	*B_G_* (**μ**m)	*D_F_* (**μ**m)	*N_n_* (1/**μ**m^2^) ×10^−3^	*A*_ CT_ (**μ**m^2^)	CT%
(1)	f	25	15443.52	453.72	158.77	7.29	2356.17	15.11
(2)	m	26	19991.87	521.23	181.76	6.32	3008.07	15.00
(3)	m	26	15834.89	470.65	164.80	7.05	2347.41	14.69
(4)	m	27	15951.55	483.49	164.75	6.19	2547.37	16.31
(5)	m	31	18635.64	513.28	180.55	6.64	4667.77	25.44
(6)	f	34	11554.94	403.31	137.86	7.96	2911.04	24.93
(7)	m	34	15356.30	459.57	164.12	7.16	2336.03	15.20
(8)	m	38	15876.03	468.91	165.19	7.34	4200.09	26.20
(9)	f	39	16646.41	478.30	163.44	6.62	3848.94	23.26
(10)	m	42	19644.56	526.75	180.53	6.09	4749.01	24.17
(11)	f	43	14188.59	439.94	153.99	7.97	3567.94	25.13
(12)	m	45	17909.25	502.77	182.01	6.32	5594.49	31.26
(13)	m	46	16756.09	485.38	169.00	6.44	5298.95	31.69
(14)	m	48	14598.70	445.02	154.80	5.95	4164.17	28.67
(15)	m	50	13349.86	451.07	151.01	6.71	3732.43	28.08
(16)	m	50	27537.67	621.61	220.90	6.48	5245.75	19.09
(17)	m	55	23605.76	580.55	196.90	5.44	8182.31	34.56
(18)	f	55	14988.72	464.78	165.75	8.03	3804.39	25.61
(19)	m	58	25780.68	589.93	204.81	5.58	8156.48	31.69
(20)	f	63	14377.20	447.77	156.59	6.30	4715.03	32.60
(21)	f	68	20900.40	537.33	188.10	6.83	7280.79	34.69
(22)	f	68	11481.99	387.06	139.79	6.63	4214.73	36.63
(23)	m	69	20727.72	542.99	185.57	4.43	6376.37	31.20
(24)	m	71	18955.20	515.81	185.54	4.93	4385.01	23.21
(25)	f	72	14407.64	452.05	160.33	6.93	4950.50	34.15
(26)	f	75	17280.41	502.18	173.87	5.57	5525.22	33.42
(27)	m	78	17235.53	499.00	181.02	7.02	6290.82	36.67
(28)	m	78	17058.38	501.24	175.88	5.53	5523.76	32.42
(29)	f	78	11506.10	399.49	151.01	7.77	4264.21	37.43
(30)	m	85	15131.32	500.46	168.95	5.94	3446.62	22.74

m: male.

f: female.

**Table 2:** Morphometric cases of the glomeruli in evaluated male and female cases.

Parameter	*A_G_* (**μ**m^2^)		*B_G_* (**μ**m)		*D_F_* (**μ**m)		*A*_ CT_ (**μ**m^2^)		CT%		*N_n_* (1/**μ**m^2^) ×10^−3^	

Variable	Mean	Md	Mean	Md	Mean	Md	Mean	Md	Mean	Md	Mean	Md
Gender	Male (*n* = 19)											

Value	18417.74	17235.54	509.46	501.24	177.79	180.53	4750.15	4667.77	25.70	26.20	6.19	6.32
SE	871.71		10.88		3.91		394.66		1.61		0.18	
95% LCL	16586.35	15834.89	486.60	470.65	169.58	164.80	3921.01	3446.62	22.32	19.09	5.82	5.58
95% UCL	20249.13	19644.56	532.32	521.23	186.00	182.01	5579.30	5523.76	29.08	31.26	6.56	6.64

Gender	Female (*n* = 11)											

Value	14797.81	14407.63	451.45	452.05	159.05	158.77	4312.63	4214.73	29.36	32.60	7.08	6.93
SE	854.52		13.54		4.31		400.92		2.11		0.24	
95% LCL	12893.83	11506.10	421.28	399.49	149.44	139.79	3419.32	2911.04	24.67	23.26	6.55	6.30
95% UCL	16701.80	16646.40	481.61	478.30	168.65	165.75	5205.94	4950.50	34.05	34.69	7.62	7.96

Md: median.

SE: standard error.

95% LCL: lower limit of confidence interval.

95% UCL: upper limit of confidence interval.

**Table 3:** Morphometric characteristics of globally sclerotic and obtained types of nonsclerosed glomeruli.

	*A_G_* (**μ**m^2^)		*B_G_* (**μ**m)		*D_F_* (**μ**m)		*A*_ CT_ (**μ**m^2^)		CT%		*N_n_* (1/*μ*m2) ×10^−3^	

Variable	Mean	Md	Mean	Md	Mean	Md	Mean	Md	Mean	Md	Mean	Md
Type	Globally sclerotic glomeruli (*n* = 114)											

Value	6851.58	6520.95	302.39	302.31	107.03	105.17	4761.58	4774.59	71.1	71.8	1.66	1.64
SE	228.52		3.85		1.44		128.66		0.5		0.05	
95% LCL	6398.84	6148.01	294.76	289.59	104.18	102.06	4506.69	4273.92	70.0	69.9	1.56	1.48
95% UCL	7304.32	7013.99	310.01	314.58	109.87	110.44	5016.48	5019.23	72.2	73.2	1.76	1.77

Type	I (*n* = 341)											

Value	16031.85	15400.52	473.34	468.55	165.13	161.62	3379.80	3296.62	21.7	21.8	6.95	6.95
SE	249.79		3.75		1.40		54.15		0.3		0.05	
95% LCL	15540.53	14563.69	465.97	452.96	162.38	158.17	3273.29	3151.55	21.1	21.0	6.85	6.83
95% UCL	16523.18	15874.55	480.71	476.86	167.88	164.44	3486.32	3437.98	22.3	22.9	7.05	7.05

Type	II (*n* = 288)											

Value	18376.17	17669.80	506.33	505.00	177.95	178.15	6018.32	5697.94	33.2	33.4	6.02	6.05
SE	323.24		5.15		1.61		104.83		0.3		0.06	
95% LCL	17739.94	16941.07	496.20	492.51	174.78	173.11	5812.00	5426.42	32.7	32.5	5.90	5.86
95% UCL	19012.39	18322.84	516.47	515.59	181.12	181.62	6224.65	5911.02	33.7	33.7	6.14	6.14

Md: median.

SE: standard error.

95% LCL: lower limit of confidence interval.

95% UCL: upper limit of confidence interval.
